# Preparation and Characterization of Activated Carbon/Polymer Composites: A Review

**DOI:** 10.3390/polym15163472

**Published:** 2023-08-19

**Authors:** Yoon-Ji Yim, Byung-Joo Kim

**Affiliations:** 1 Busan Textile Materials Research Center, Korea Dyeing and Finishing Technology Institute, Busan 46744, Republic of Korea; yjyim@dyetec.or.kr; 2 Department of Nano & Advanced Materials Engineering, Jeonju University, Jeonju 55069, Republic of Korea

**Keywords:** activated carbon, polymer composites, composite preparation, composite properties

## Abstract

Activated carbon (AC) and activated carbon fibers (ACFs) are materials with a large specific surface area and excellent physical adsorption properties due to their rich porous structure, and they are used as electrode materials to improve the performance of adsorbents or capacitors. Recently, multiple studies have confirmed the applicability of AC/polymer compo-sites in various fields by exploiting the unique physical and chemical properties of AC. As the excellent mechanical properties, stability, antistatic and electromagnetic interference (EMI) shielding functions of activated carbon/polymer composite materials were confirmed in recent studies, it is expected that activated carbon can be utilized as an ideal reinforcing material for low-cost polymer composite materials. Therefore, in this review, we would like to describe the fabrication, characterization and applicability of AC/polymer composites.

## 1. Introduction

Activated carbon (AC) and activated carbon fibers (ACFs) have rich porous structures, large specific surface areas, and excellent physical adsorption properties. Such AC and ACFs can be synthesized using various raw materials such as coal [1], petroleum [2], biomass [3], and the like, and activated carbon having various properties can be manufactured according to activation methods such as gas activation [4] and chemical activation [5]. Usually, owing to its excellent adsorption performance, AC is widely used in all industries as an adsorbent for decolorization, deodorization, solvent recovery, and water and wastewater treatment; furthermore, it has been used as an electrode material to improve the performance of capacitors [6,7,8,9,10,11,12,13,14,15,16,17,18,19,20,21,22,23,24,25,26,27,28,29].

As it is applied to various fields, the demand in the market has increased widely. In addition, recently, research on using AC and ACFs as a filler for the development of high-functional polymer composite materials [30,31,32,33,34,35,36,37,38,39,40,41,42,43,44,45,46,47,48,49] has been steadily progressing just like carbon materials such as carbon fibers [50,51,52,53,54], carbon nanotubes [55,56,57,58,59,60,61,62,63,64,65,66,67,68], and graphene. However, AC and ACFs have great potential to replace other carbon materials, but their potential use in polymer composite materials as reinforcing fillers has not been fully explored.

Recently, multiple studies have confirmed the applicability of AC/polymer composites in various fields by exploiting the unique physical and chemical properties of AC. For example, due to the porous structure, the molten polymer enters the pores of the activated carbon to form a three-dimensional network to improve the tensile properties of the composite material, or to improve the electrical properties or electromagnetic wave shielding properties of the polymer composite material due to its electrical conductivity. Owing to its low cost, excellent stability, and antistatic and electromagnetic interference shielding functions, AC/polymer composite material can be used in various applications, such as automobile parts and shielding materials [69,70,71,72,73,74,75,76,77,78,79,80,81,82,83,84,85,86,87,88,89,90,91,92,93,94,95,96,97,98].

This work aimed to investigate the current status of related research fields by summarizing the manufacturing, characterization, and applicability of AC/polymer composite materials reported in the literature. Herein, the applicability and properties of AC/polymer composite materials, such as adsorption, mechanical, electromagnetic interference shielding (EMI SE), and other properties, according to various manufacturing conditions are detailed.

## 2. Adsorption Properties of Activated Carbon/Polymer Composites

**Figure 1:**
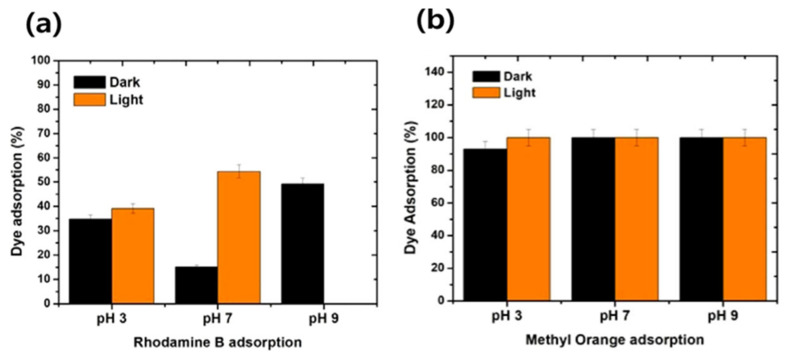
Dye adsorption efficiency of (**a**) RhB cationic dye and (**b**) methyl orange anionic dye [71]. “Reprinted/adapted with permission from Ref. [71]. 2020, John Wiley and Sons”.

Various studies have been conducted on the evaluation of the absorption properties of AC/polymer composites; the results of some studies are summarized in Table 1. Akter et al. [69] investigated the removal properties of Pb(II) using chitosan-activated carbon-polyvinyl alcohol (CS-AC-PVA) composite beads and reported that the amount of Pb adsorption was 0.2801 mg/g. Bekhoukh et al. [70] used an activated carbon/polyaniline composite as an adsorbent to confirm the properties of anionic methyl orange removal. Ramadoss et al. [71] used an activated carbon/PVP composite to fabricate a biodegradable membrane for brackish water treatment; the results indicated that the dye adsorption efficiency was 100% for methyl orange and up to 57% for rhodamine B within 3 h (Figure 1). 

Lelifajri et al. [72] prepared an immobilized activated carbon/polyvinyl alcohol composite material for the adsorption removal of 2,4-dichlorophenoxyacetic acid. The results indicated that the prepared adsorbent exhibited adequate reuse performance and that it was an effective and promising adsorbent for removing herbicides. Industrial wastewater with heavy metals is a major environmental problem, and research on the development of adsorbents that can remove these heavy metals is ongoing. Aswini and Jaisankar [73] investigated the adsorption of heavy metals, such as copper, cadmium, and lead, using sugarcane-based AC and polymer composites. The results confirmed that the prepared AC and composite material can be used as an adsorbent for the removal of heavy metal in wastewater. Khalili et al. [74] synthesized a pine cone-based activated carbon/polyaniline composite for capturing CO_2_ via oxidative polymerization and analyzed the CO_2_ adsorption capacity of the prepared composite. The results indicated that the CO_2_ adsorption capacity significantly increased from 1.91 mmol/g for AC to 2.69 mmol/g for AC-PANI-F and 3.16 mmol/g for AC-PANI-S at 25 °C and 1 bar. This indicates that activated carbon/PANI composites can be used as effective adsorbents to capture CO_2_ from flue gases. Hwang et al. [75] studied the properties and filtration efficiency of activated carbon/polymer composites for humic acid removal. The results indicated that AC addition significantly affected the membrane morphology, pore size distribution, porosity, and chemical properties. Activated carbon/polyurethane foam composites were prepared by adding AC during PU foam synthesis. The prepared composite material exhibited the maximum adsorption capacities of MB and phenol of 100 and 66.5 mg/g, respectively. The manufactured AC/PU composite material can be molded into a desired shape depending on the mold used, confirming its applicability in various fields [76].

## 3. Mechanical Properties of Activated Carbon/Polymer Composites

**Figure 2:**
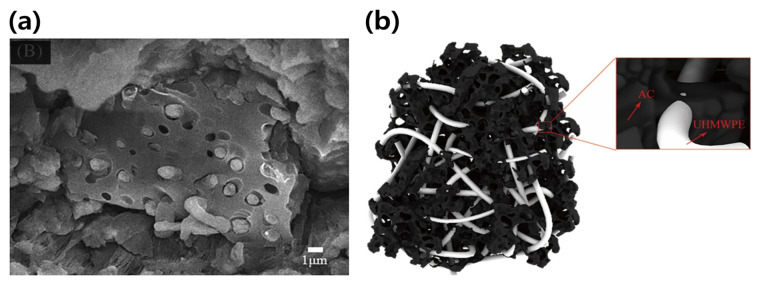
Schematic diagram of the internal bonding model of AC/polymer composites (**a**) SEM image of the tensile section of AC/UHMWPE composite material (4000×), (**b**) Schematic diagram of the internal bonding model of AC/UHMWPE composite material [77]. “Reprinted/adapted with permission from Ref. [77]. 2021, John Wiley and Sons”.

Many studies have been conducted on the evaluation of the mechanical properties of AC/polymer composites; the results of some studies are summarized in Table 2. Wang et al. [77] reported the manufacturing method and analyzed the mechanical properties of biomass-activated carbon (BAC)/ultra-high-molecular-weight polyethylene (UHMWPE) composites using physical mixing and the twin-screw extrusion process. The results indicated that when 65% of BAC was added, the tensile strength increased by approximately 325.86%, from 22.93 to 97.65 MPa. The uniformly distributed BAC in the composites improved the mechanical properties. They explained that the molten UHMWPE entered the pores of the BAC powder and formed a three-dimensional network through pore bonding (Figure 2a,b), effectively bearing the load and improving the tensile properties of the composites. 

It was confirmed through the SEM images that the molten UHMWPE was embedded in the pores of the BAC like a rivet structure. Nisar et al. [78,79] prepared a polyethylene (PE) nanocomposite reinforced with magnetic (Ni, Co, Fe)-AC using the melt mixing method and investigated the mechanical properties of the composites. The results confirmed that the AC-Ni/PE composites showed the highest improvement in mechanical properties, and the modulus of the composites was 1202 MPa, which was approximately 53% higher than that of HDPE. They explained that the elongation at break decreases by strongly limiting the strength of the polymer chain as the fillers are dispersed in the polymer matrix, and they also showed the improvement of mechanical properties through various factors such as the dispersion of the filler and the crystal structure of the polymers.

**Figure 3:**
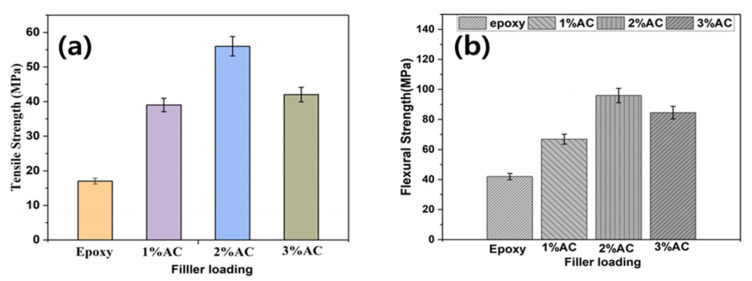
(**a**) Tensile strength of AC/epoxy composites as a function of filler loading, (**b**) Flexural strength of AC/epoxy composites as a function of filler loading [80]. “Reprinted/adapted with permission from Ref. [80]. 2020, John Wiley and Sons”.

Minugu et al. [80] investigated the effect of AC content on the mechanical strength of composite materials using Arhar fiber biomass as a precursor and a reinforcing material for synthesized AC epoxy composites. Minugu’s research team prepared AC with many micropores and mesopores, and they confirmed that the strength of the composites increases as the polymer resin penetrates the porous structure of AC and forms a strong bond. The results indicated that the tensile and flexural strengths of the composite material with 2% AC were 56 and 95 MPa, respectively (Figure 3a,b). 

Makara et al. [81] developed a technique for converting dead leaf biomass into AC and studied the mechanical properties of the composites using it as a reinforcing material for natural rubber. For comparison with carbon black (CB), which is generally used as a reinforcing material for rubber, the mechanical properties of CB/rubber and AC/rubber composite were comparatively analyzed. Both types of carbon fillers improved the mechanical strength of the rubber, which was explained by the network formation caused by the interaction between the filler and rubber. The tensile strength and modulus of the composite with 15 PHR of dead leaf-AC increased by ~8% and 40%, respectively. The CB/rubber composites had better mechanical properties than the AC/rubber composites. However, in the case of the studied dead leaf-AC, it was confirmed that it is a promising material that can improve the mechanical properties of rubber at a lower price than CB. Mustafa et al. [82] investigated the tensile strength of an epoxy composite material according to the AC content. The AC content of each composite was 0, 5, 10, 15, 20, 25, 30, 35, and 40 wt.%, and the composite with AC of 15 wt.% had the best tensile strength. The results indicated that the tensile strength of the composite material with 15 wt.% of AC was 36.34 MPa, which is an improvement of approximately 19%. They also demonstrated through FT-IR, SEM, DSC analysis, etc. that there is a strong interaction between the epoxy matrix and AC powder. Nawras et al. [83] investigated the effect of the addition of AC on the mechanical properties of jute fiber/polyester composites. The AC content of each composite was 1,3,5, and 10 wt.%, and the mechanical properties also improved as the AC content increased. The composites with 3 wt.% AC had the best impact strength, and when 3 wt.% AC was added, the impact strength increased by approximately 51% to 6.4 kJ/m^2^. Mahmud et al. [84] investigated the effects of contact pressure and sliding speed on the friction coefficient and wear of activated carbon/epoxy composites. Hilmi et al. [85] investigated the frictional properties of activated carbon/epoxy composites. Wang and Su [86] investigated the effect of AC surface treatment on the mechanical properties of epoxy composite materials and confirmed that the fracture toughness of the composite material improved by approximately 234%. They explained that the reinforcing mechanism of mechanical properties is due to the physical properties of AC due to the large specific surface area and porous structure and the chemical bond formed by the reaction of the functional group of AC with the epoxy group of matrix. Khalil et al. [87] investigated the fracture toughness of an activated carbon/epoxy composite material with an addition of 5% AC and reported that the fracture toughness of the composite material improved by approximately 17%. Song et al. [88] confirmed the compressive strength of a specimen by manufacturing AC/phenolic foam. The AC/phenol was foamed using microwaves. They explained that AC strengthens the foam structure by trapping gases such as H_2_O generated during the curing reaction. The compressive strength of the specimen with AC was approximately 9.7% higher than that of the specimen without. These showed that the addition of AC and AC surface treatment are effective at improving the mechanical properties of composite materials. Most of the research results showed that the mechanical properties of the AC/polymer composites were improved as the polymer penetrates the pores of the AC and the bonding force between the two materials increases. These research results confirmed the improvement of the mechanical properties of the composites by adding AC, suggesting the possibility of application in various fields such as automobiles and aviation parts.

## 4. Electrical and EMI Shielding Properties of Activated Carbon/Polymer Composites

**Figure 4:**
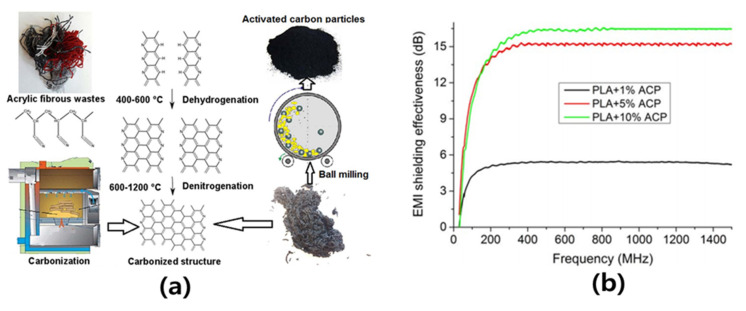
(**a**) Preparation of activated carbon from acrylic fibrous waste and (**b**) EMI shielding effectiveness of AC/PLA composites [91]. “Reprinted/adapted with permission from Ref. [91]. 2019, John Wiley and Sons”.

Some research results on the Electrical and EMI shielding properties of AC/polymer composites are summarized in Table 3. Singsang et al. [89] mixed AC synthesized from waste coffee grounds with poly(butylene succinate) (PBS) in a batch-type internal mixer and confirmed the influence of AC content on physical properties. They prepared the AC/PBS composites by adjusting the concentration of AC to 0, 1, 1.5, 2 and 5 wt.%, and they investigated their mechanical, electrical, and morphological properties. The electrical properties of the composites were confirmed through electrical conductivity measurement, and it was confirmed that the electrical conductivity of the composites increased rapidly when 1.5 wt.% of AC was added. The electrical properties of the AC (5 wt.%)/PBS composites were the best. They explained that it was due to the high electrical conductivity of AC. The electrical conductivity of the composite material with 5% activated carbon was 4.32 × 10^−5^ (Ω m)^−1^, which was approximately 229% higher than that of PBS, confirming that AC could be used as a reinforcement in the PBS matrix. Abdullah et al. [90] prepared the Fe-AC powders by chemically bonding Fe to AC, and they confirmed that a stable bond was formed through analysis such as FT-IR. In addition, adjusting the content of the Fe-AC powders to 10, 20, and 25% to prepare Fe-AC/PVA composites confirmed the electromagnetic properties. In particular, they confirmed the electromagnetic wave absorption properties of the Fe-Ac/PVA composites; the results indicated that this composite material exhibits excellent electromagnetic wave absorption properties in the frequency range of 4–6 GHz and that the composite material with 20% AC has a return loss value of −32.5 dB at a frequency of 4.65 GHz. Naeem et al. [91] reported research results for the development of multifunctional PLA composites by synthesizing AC from acrylic fiber waste (Figure 4). Acrylic fibrous waste–AC particles were added to PLA at 1, 5, and 10 wt.% to prepare AC/PLA composite films by solvent casting, and their electrical conductivity, EMI shielding, thermomechanical and thermal stability properties were investigated. The electrical conductivity of the acrylic fibrous waste–AC/PLA composite films was observed by measuring the surface resistance, and as a result of the analysis, it was confirmed that the resistance decreased rapidly after adding 10 wt.% to the acrylic fibrous waste–AC. They explained that this was due to the tunneling effect. In addition, the analysis of the EMI shielding properties of the activated carbon/PLA composite revealed a shielding effect of approximately 16 dB, confirming its potential as a packaging material for electronic devices.

**Figure 5:**
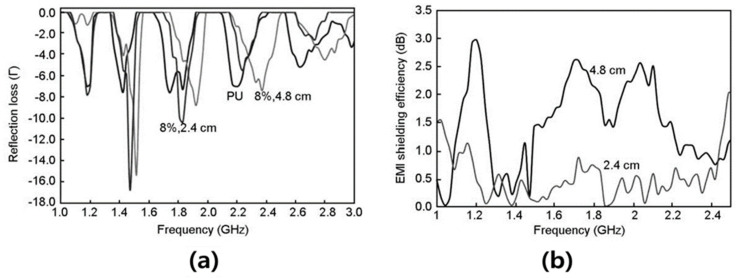
(**a**) Return loss spectrum of different thicknesses of 8% R1.5 loaded PU foam at the frequency range of 1 to 3 GHz and (**b**) EMI shielding efficiency spectra of different thicknesses of 8% R1.5-loaded PU foam at the frequency range of 1 to 2.5 GHz [93]. “Reprinted/adapted with permission from Ref. [93]. 2015, Elsevier”.

Xia et al. [92] manufactured an activated carbon/kenaf fiber/polyester composite using the VARTM process and investigated the electromagnetic wave shielding properties of the composite material. The AC content of the composite material was added as 0, 10, 20, and 30%, and the EMI shielding properties of the composite material were confirmed according to the AC content. With increasing AC content, the EMI shielding effectiveness increased to 93.0%. The incorporation of AC into the composite was very effective at shielding electromagnetic waves. They explained that the addition of AC is particularly effective in enhancing the electromagnetic wave absorption of the composite, which reduces secondary electromagnetic pollution. Shaaban et al. [93] manufactured an AC (8 wt.%)/PU composite material using the chemical blowing method and investigated its microwave absorption characteristics. They synthesized a novel AC using rubber wood sawdust as a raw material, and the prepared AC had a BET surface area of 1301 m^2^/g. As the AC content increased, the electromagnetic wave shielding properties increased, and the AC (8 wt.%)/PU composites showed the best shielding properties. The results of EMI shielding of AC (8 wt.%)/PU indicated that it has a value of approximately 10 dB (Figure 5). Khan et al. [94] prepared an AC/acrylic resin (AR) composite material according to the AC content using the solution processing method. The AC/AR composites were prepared with AC content of 0, 1, 5, 10, 20, and 30%, and the composites were characterized through FT-IR, XRD, TGA, and SEM analysis. In addition, electrical properties were measured to confirm the applicability of the AC/AR composite material as an electromagnetic wave shielding material. The electrical properties of the AC/AR composites were confirmed by measuring the surface resistance. With increasing AC content, the electrical resistivity of the composite material decreased. As the AC content increased, the electrical resistance of the composite material decreased. The composite material with 30% of AC had a considerably lower resistivity (10^4^ ohm/sq) compared to that of the pure acrylic resin. These results suggest that activated carbon/acrylic composites are suitable materials for electromagnetic wave shielding coatings.

Junhua and Chung [95] confirmed the electromagnetic wave shielding properties of AC fiber/carbon fiber/polymer composite materials; the electromagnetic wave shielding effect of these materials was 38 dB, which was approximately 30% higher than that of the carbon fiber/epoxy composite material. Khan et al. [96] prepared a composite by dispersing AC (1~30 wt.%) in an acrylic resin (AR) matrix and confirmed the electromagnetic shielding properties of the composites. In addition, they analyzed the structural and morphological properties of AC/AR composites through XRD, Raman, and SEM analysis, and they confirmed that AC was effectively dispersed inside the AR matrix. It was confirmed that the electromagnetic wave shielding effect value of the AC (30 wt.%)/AR composite material was −36 dB, and this value corresponds to about 1400% of the AR shielding value (−2.4 dB). As a result of the study, the AC/AR composites had a shielding efficiency of 99.9% or more, confirming that it is a suitable material as an EMI shielding material. Yew et al. [97] prepared a composite by dispersing a hybrid filler containing AC in an epoxy resin matrix. Three types of fillers were used: coconut shell (CS), CS-AC, and beta-silicon carbide (β-SiC). The elemental composition, surface morphologies and structural properties of epoxy composites according to hybrid fillers were investigated, and it confirmed that CS and CS-AC fillers had a positive effect on the electromagnetic properties of composite materials from the results of dielectric properties and electrical conductivity analysis.

## 5. Other Properties of Activated Carbon/Polymer Composites

Table 4 shows the research results on other properties of AC/Polymer composites. Yun et al. [98] prepared a composite hydrogel by adding AC to poly(vinyl alcohol) (PVA) and poly(acrylic acid) (PAAc) polymers by free-radical polymerization. The PVA/PAAc composite hydrogels containing two different types of AC, coconut-based AC and coal-based AC, were prepared. The drug was efficiently loaded due to the abundant pore structure of AC in the fabricated composite hydrogel system. The hydrogel matrix used a swelled well in basal conditions to release the drug loaded into the AC. The drug-release behavior of AC/PVA/PAAc composite materials was studied, and it was confirmed that the composite hydrogel containing AC by changing the type and pH of AC is a material that can easily control drug release.

**Figure 6:**
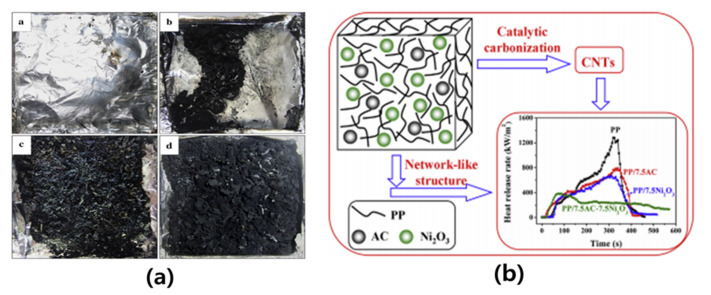
(**a**) Photographs of the residual chars after the cone calorimeter tests from neat PP (a), PP/7.5AC composite (b), PP/7.5Ni_2_O_3_ composite (c) and PP/7.5ACe7.5 Ni_2_O_3_ composite (d) and (**b**) Schematic drawing for the mechanism of the synergistic effect between AC and Ni_2_O_3_ in improving the flame retardancy of PP [99]. “Reprinted/adapted with permission from Ref. [99]. 2014, Elsevier”.

Gong et al. [99] prepared AC/Ni_2_O_3_/polypropylene composites through melt mixing and investigated the thermal stability and flame retardancy of the composites. They confirmed that AC was effectively dispersed in the PP matrix through XRD, SEM, and TEM analysis, and they studied thermal stability through thermogravimetric analysis and flame retardancy through cone calorimeter testing. The addition of AC/Ni_2_O_3_ significantly improved the thermal stability of the PP composites and significantly reduced the heat release rate. As a result of the study, it was confirmed that the thermal stability and flame retardancy of the composite material were improved by forming a network structure of AC and Ni_2_O_3_ particles in the matrix. They explained that the formation of a denser protective layer due to the formation of a network of AC/Ni_2_O_3_ particles in the PP matrix improved the flame retardancy of the PP composites (Figure 6).

Alston et al. [100] characterized the hygroscopic behavior of ACFs/phenolic resin composites, and as a result of the study, it was confirmed that activated carbon fibers act as sinks or water sources by absorbing water due to pores. The observed water absorption and diffusion behavior of the ACFs/phenolic resin composites was very complex due to the combination of water adsorbed and absorbed by the fibers and water in the resin due to curing. They have successfully developed a “source-sink” model to simulate the absorption and diffusion behavior of the composites. In addition, the porosity of the ACFs confirmed that a high level of accuracy could be achieved using the proposed “source sink” extension model. Zhang et al. [101] prepared polyvinyl chloride (PVC) composites containing AC and molybdenum oxide (MoO_3_), and they investigated the effect of the addition of AC and MoO_3_ on improving the flame retardancy of the composite. As a result of the analysis, it was confirmed that as AC and MoO_3_ were added, the heat dissipation property of the composite material was greatly reduced, and the smoke suppression property was greatly improved. When the total content of AC and MoO_3_ was 10 PHR, the flame retardancy of the composite material was the best, and the heat release rate and smoke generation rate values were the lowest at 173.80 kW/m^2^ and 0.1472 m^2^/s, respectively. This is a result of 47.3% and 59.9% reduction, respectively, when compared with the value of PVC. Oh et al. [102] prepared a composite material by adding AC to a mixture of poly(ε-caprolactone) (PCL) and poly(ethylene oxide) (PEO) polymers, and the drug release properties of AC/PCL/PEO composites were investigated. As a result of the analysis, the drug release properties of the AC/PCL/PEO composites were significantly improved due to the microporous structure of AC and the swelling and selective dissolution properties of the PCL/PEO polymers. In addition, the prepared composites were effective in controlling both drug loading and drug release. The study results of the drug release properties, thermal stability, flame retardancy, and hygroscopicity were summarized as other properties of the AC/polymer composites. Through this, it was possible to confirm the application potential of the AC/polymer composite material for drug delivery as well as flame-retardant and moisture-absorbent materials, and the possibility of application in various other fields can also be expected.

## 6. Conclusions

Through this work, the current status of related research fields is investigated in order to confirm the preparation, characterization, and applicability of AC/polymer composites reported in the literature. This review summarizes the results of studies on the effects of AC and ACFs as reinforcing materials in polymer composites on the mechanical properties, adsorption properties, and electrical properties of the composite materials. AC and ACFs are materials with a rich porous structure, a large specific surface area, and excellent physical adsorption properties. Using the unique physical and chemical properties of AC, it is possible to develop AC/polymer composites for application in various fields. As the polymer matrix penetrates the pores of AC, the mechanical properties of AC/polymer composites are significantly improved. The dispersion properties and surface properties of AC are also important factors in determining the mechanical properties of composites. Due to the excellent adsorption properties of AC, AC/polymer composites can be used as adsorbents for dyes and heavy metals. In addition, AC presents the possibility of application as an electromagnetic wave shielding material and an antistatic material by giving electrical properties to the polymers. The application potential of the AC/polymer composite material for drug delivery, flame retardant, and moisture absorption was also confirmed. The research results summarized in this review show positive effects of AC and ACFs added to polymer composites, confirming their potential use in various industrial applications such as aerospace, EMI shielding, automotive, and sporting goods. In addition, activated carbon is a very inexpensive material compared to nano-carbon materials such as CNTs and graphene, suggesting the possibility of developing a low-cost, high-functional composite material through various types of activated carbon and various surface treatment technologies. Through various research results, the possibility of application in other various fields of AC/polymer composite can also be expected.

## Figures and Tables

**Table 1:** Adsorption characteristics of AC/polymer composites according to the preparation condition.

Preparation Condition	Adsorption Characteristics	Enhancement
Chitosan (3 g)-AC (1 g)/PVA (4 g) composites by mixing and heating [69]	Adsorbed amount of Pb (0.2801 mg/g)	Adsorbed amount of Pb: 115.95% (compared with CS)
AC/PANI composites via in situ polymerization [70]	MO removal capacity (192.52 mg g^−1^ at 298 K and pH 6.0)	311.19% (compared with PANI)
AC/NaCMC/PVP (1:3:2) composites using the solution casting method [71]	Adsorption of two toxic dyes, Rhodamine B (57%) and methyl orange (100%)	-
AC/polyvinyl alcohol composites using the solution method [72]	Adsorption of 2,4-dichlorophenoxyacetic acid (55.9 mg/g)	-
Sugarcane bagasse–AC/PVP composites [73]	Adsorption capacity of Pb (96.39%), Cu (98.38%), and Cd (79.43%)	4.63%, 3.15%, and 2.75% (compared with AC)
AC/polyaniline composites via in situ polymerization [74]	CO_2_ adsorption capacity (3.16 mmol/g)	65.44% (compared with AC)
AC/PPSU/PEI/PEG (0.25/35/5/6 wt.%) composites using the wet phase inversion technique [75]	Humic acid removal efficiency (80%)	-
AC/polyurethane foam (PU) composites [76]	Adsorption capacity of MB (100 mg/g)	-

**Table 2:** Mechanical characteristics of AC/polymer composites according to the preparation condition.

Preparation Condition	Mechanical Characteristics	Enhancement
BAC (65%)/UHMWPE (35%) composites using the twin-screw extrusion process [77]	Tensile strength (97.65 ± 5.23 MPa)	Tensile strength: 325.86% (compared with UHMWPE)
AC–Ni/PE composites (2 wt.%) composites using the melt mixing technique [78,79]	Tensile modulus (1202 MPa)	Tensile modulus: 53.51% (compared with HDPE)
AC (2%)/epoxy composites using mechanical stirring methods [80]	Tensile and flexural strengths (56 and 95.2 MPa, respectively)	Tensile strength: 329% Flexural strength: 226% (compared with epoxy)
Dead leaf-AC (15 PHR)/rubber (SMR) composites using the mixing and compounding process [81]	Tensile strength and modulus elongation	-
AC (15 wt.%)/epoxy composites using a laboratory shear mixture [82]	Tensile strength (26.34 MPa)	Tensile strength: 19.16%
AC (3%)/jute fiber (21%)/polyester composites using the hand lay-up process [83]	Impact strength (6.4 kJ/m^2^)	Impact strength: 51%
AC (60%)/epoxy (40%) composites using a hot-press machine [84]	Coefficient of friction and wear	-
PKAC (70 mass%)/epoxy (30 mass%) composites using a hot-press machine [85]	Friction coefficient	-
0.3% NAC (ammonia-treated)/epoxy composites using mill technology [86]	Fracture toughness (*K*_IC_: 3.88 ± 0.06 MPa m^1/2^)	*K*_IC_: 234.48%
AC (5%)/epoxy composites by mixing [87]	Fracture toughness (0.92 J)	Fracture toughness: 17.94%
phenolic resin (90)/acid (10)/AC (1) composite foams by microwave foaming [88]	Compressive strength (2170 kPa)	Compressive strength: 9.7%

**Table 3:** EMI shielding characteristics of AC/polymer composites according to the preparation condition.

Preparation Condition	Electrical and EMI Shielding Properties	Enhancement
Coffee-AC (5 wt.%)/PBS composites using the melting process [89]	Electrical conductivity (4.32 × 10^−5^ (Ω·m)^−1^)	229.77% (compared with PBS)
Fe-AC (20%)/PVA composites using the solution method [90]	Microwave absorption (−32.5 dB)	-
Acrylic fibrous waste–AC (10 wt.%)/PLA composites via solvent casting [91]	EMI shielding properties (16 dB) and electrical conductivity (10,000 Ω·cm)	EMI shielding properties: 220% (compared with AC 1 wt.%) Electrical conductivity: 5 × 10^15^ Ω·cm (Pure PLA)
AC (28.9%)/polyester composites using the VARTM process [92]	EMI shielding properties (93%)	124.63% (compared with AC 0%)
AC (8 wt.%)/PU composites using the chemical blowing method [93]	Microwave absorption (10 dB)	-
AC (30 wt.%)/acrylic resin(AR) composites using the solution process [94]	Electrical resistivity (10^4^ Ω/sq)	Pure AR (10^11^ Ω/sq)
ACF/epoxy composites using filament winding machine [95]	EMI shielding properties (38 dB)	31% (compared with CF/epoxy)
AC (30 wt.%)/acrylic resin(AR) composites using the solution process [96]	EMI shielding effectiveness (−36 dB)	1400% (compared with AR)
Coconut shells(CS)/CS-AC/beta-silicon carbide (β-SiC)/epoxy resin composites using the solution process [97]	Dielectric properties and Electrical conductivity	-

**Table 4:** Other characteristics of AC/polymer composites according to the preparation condition.

Preparation Condition	Other Properties	Enhancement
AC/PVA/PAAc composite hydrogel by free-radical polymerization [98]	Drug release behavior	-
AC/Ni_2_O_3_/polypropylene composites using the melt mixing process [99]	Thermal stability and flame retardancy	-
ACFs/phenolic resin composites composites via solvent casting [100]	Hygroscopic behavior	-
AC/MoO_3_/PVC composites using the solution process [101]	Heat release rate (173.80 kW/m^2^) and smoke generation rate (0.1472 m^2^/s)	47.3% and 59.9% reduction (compared with PVC)
AC/PCL/PEO composites using the oil-in-water emulsion solvent evaporation method [102]	Drug release behavior	-

## Data Availability

This article is a review article, so there is no data to share.
